# Genome-wide screening in human embryonic stem cells identifies genes and pathways involved in the p53 pathway

**DOI:** 10.1186/s10020-025-01141-5

**Published:** 2025-03-13

**Authors:** Amir Haddad, Tamar Golan‐Lev, Nissim Benvenisty, Michal Goldberg

**Affiliations:** 1 Department of Genetics, The Alexander Silberman Institute of Life Sciences, the Hebrew University of Jerusalem, 91904 Jerusalem, Israel; 2 The Azrieli Center for Stem Cells and Genetic Research, The Alexander Silberman Institute of Life Sciences, the Hebrew University of Jerusalem, 91904 Jerusalem, Israel

**Keywords:** CRISPR-cas9 screening, p53 pathway, Nutlin-3a, TRIP12

## Abstract

**Background:**

The tumor suppressor protein, p53, which is mutated in half of human tumors, plays a critical role in cellular responses to DNA damage and maintenance of genome stability. Therefore, increasing our understanding of the p53 pathway is essential for improving cancer treatment and diagnosis.

**Methods:**

This study, which aimed to identify genes and pathways that mediate resistance to p53 upregulation, used genome-wide CRISPR-Cas9 loss-of-function screening done with Nutlin-3a, which inhibits p53-MDM2 interaction, resulting in p53 accumulation and apoptotic cell death. We used bioinformatics analysis for the identification of genes and pathways that are involved in the p53 pathway and cell survival assays to validate specific genes. In addition, we used RNA-seq to identify differentially expressed p53 target genes in gene knockout (KO) cell lines.

**Results:**

Our screen revealed three significantly enriched pathways: The heparan sulfate glycosaminoglycan biosynthesis, diphthamide biosynthesis and Hippo pathway. Notably, *TRIP12* was significantly enriched in our screen. We found that *TRIP12* is required for the p53-dependent transcription of several pro-apoptotic genes.

**Conclusion:**

Our study has identified two novel pathways that play a role in p53-mediated growth restriction. Moreover, we have highlighted the interaction between the Hippo and the p53 pathways. Interestingly, we have shown that *TRIP12* plays an important function in the p53 pathway by selectively affecting its role as a transcription factor.

**Supplementary Information:**

The online version contains supplementary material available at 10.1186/s10020-025-01141-5.

## Introduction

p53, a protein encoded by the *TP53* gene, is a tumor suppressor protein involved in the cellular response to a diverse set of cellular stresses, such as DNA damage, oncogene activation and hypoxia (Wang et al. 2010; Beckerman and Prives 2010; Niazi et al. 2018). It mainly acts as a transcription factor, that orchestrates many cellular responses, such as apoptosis, DNA damage repair and cell cycle arrest (Wang et al. 2010; Beckerman and Prives 2010; Niazi et al. 2018; Fischer 2017). Consequently, this protein is widely regarded as “the guardian of the genome”. *TP53* is the most frequently mutated gene in human cancer (Hainaut and Pfeifer 2016), with mutations occurring in approximately half of human tumors (Vousden and Lu 2002). In addition, germline mutations of the *TP53* gene are the result in Li-Fraumeni syndrome, which predisposes carriers to the development of a wide variety of cancers (Guha and Malkin 2017).

p53’s robust role in tumorigenicity makes it a potential target for cancer therapy. For example, small molecule inhibitors targeting the MDM2-p53 protein–protein interactions, such as Nutlin-3a (hereby referred to as Nutlin), have been developed to stabilize p53 (Vassilev et al. 2004). MDM2, an E3 ubiquitin ligase, is the most prominent negative regulator of p53 (Vousden and Lu 2002). Therefore, inhibiting the interaction between MDM2 and p53 in tumors containing wild type (WT) p53 stabilizes p53, leading to large scale apoptosis and potentially abolishing the tumor.

When activated, p53 regulates downstream genes that can lead the cell to different fates. Among the most prominent of these genes is *CDKN1A*, which encodes the p21 protein. p21 is a cyclin-dependent kinase inhibitor that directly interacts with CDK complexes, leading to their inhibition and ultimately causing cell cycle arrest (Xiong et al. 1993; El-Deiry et al. 1994). p21 has the highest affinity to cyclin E/CDK2 and cyclin D/CDK4, leading to G1 arrest (Harper et al. 1995). 14–3-3σ and Gadd45 are both transcriptionally activated by p53 and can induce G2/M arrest, with Gadd45 also capable of causing G1/S arrest (Wang et al. 1999; Hermeking et al. 1997; Kastan et al. 1992). p53 also regulates apoptotic genes, such as the death receptors FAS and DR5, leading to apoptosis (Sheikh and Fornace 2000). Moreover, p53 activates the proapoptotic proteins Bax, Noxa, Puma and BID, which ultimately lead to the release of cytochrome C from the mitochondria and the activation of caspases (Miyashita et al. 1994; Michalak et al. 2008; Sax et al. 2002; Chipuk et al. 2004).

In addition to its transcriptional activity, p53 regulates translation both globally and on a gene-specific level (Marcel et al. 2015). This regulation is emerging as an important branch of p53 function. p53 can control the translation of specific mRNAs by binding 5’UTR of various mRNAs, such as CDK4 and FGF2 (Miller et al. 2000; Galy et al. 2001). Additionally, p53 can affect the global rate of translation by modulating the activity of RNA polymerases I and III, thereby affecting the production of ribosomes and tRNAs (Chesnokov et al. 1996; Zhai and Comai 2000). Moreover, p53 directly interacts with 5.8S rRNA and eukaryotic elongation factor 2 (eEF2) (Fontoura et al. 1992; Yin et al. 2003; Argüelles et al. 2013).

Loss of function (LoF) genetic screening has become a popular approach for studying various molecular mechanisms involved in a wide array of cellular phenotypes (Yilmaz et al. 2018; Shalem et al. 2015). For example, genetic screening can be used to investigate genes responsible for chemotherapy resistance (Wei et al. 2019; Segal et al. 2023). Among the various LoF methods, CRISPR-Cas9 knockout screens provide the most reliable and efficient results (Evers et al. 2016). We have previously generated a genome-wide CRISPR-Cas9 LoF library targeting 18,166 protein-coding genes, prepared using karyotypically normal haploid human embryonic stem cells (hESCs) isolated in our lab (Yilmaz et al. 2018; Sagi et al. 2016a). Using haploid cells in CRISPR-Cas9 screens abolishes the noise produced by heterozygous cells, making it more reliable than using diploid cells. Furthermore, the use of hESCs, which are genetically stable in comparison with cancer cell lines, allows us to produce robust results that are less affected by the high probability noise associated with the genomic instability of cancer cells. We utilized our haploid CRISPR library cells to construct an atlas of essential and growth-restricting genes in hESCs (Yilmaz et al. 2018).

In this study, we utilized this library to study p53 interactors by treating the cells with Nutlin, which is toxic to cells as it increases p53 levels. We identified genes that enabled the cells to evade p53-mediated apoptosis and growth inhibition, and hence were enriched in our screen. We demonstrate that the inhibition of the heparan sulfate (HS) glycosaminoglycan biosynthesis, diphthamide biosynthesis and Hippo pathway facilitate the bypass of these p53-mediated responses. Moreover, *TRIP12* was among the highly enriched genes in our screen. Further investigation showed that this is possibly achieved by affecting the transcription of p53 canonical target genes in the absence of *TRIP12*.

## Methods

### Cell culture

Haploid hESC (h-pES10 cell line (Sagi et al. 2016a)) CRISPR-Cas9 library cells were cultured on feeder-free 10 cm (Corning, Glendale, AZ, USA) Matrigel (BD Biosciences, Le Pont-de-Claix, France) coated plates using mTeSR medium (STEMCELL Technologies, Vancouver, BC, Canada) supplemented with 50 µg/ml streptomycin, and incubated at 37 °C and 5% CO_2_. In addition, 10 μM ROCK inhibitor Y-27632 (Stemgent, Boston, MA, USA) was supplemented during thawing/passaging of cells for the first 24 h. The cells were passaged by trypsinization using trypsin–EDTA (Biological Industries, BI, Kibbutz Beit Haemek, Israel) and plated as described above.

Haploid hESCs grown on feeder layer growth-arrested mouse embryonic fibroblasts (MEFs) were grown using standard hESC growth medium composed of knockout Dulbecco’s modified Eagle’s medium (DMEM), with 2 mM l-glutamine, 0.1 mM β-mercaptoethanol, 50 μg/ml streptomycin, 50 units/ml penicillin, 0.1 mM nonessential amino acids, 8 ng/ml basic fibroblast growth factor (bFGF) and 15% knockout serum replacement (Thermo Fisher Scientific, Paisley, UK). MEFs and 293 T cells were grown using DMEM supplemented with 10% fetal bovine serum (Biological Industries), 2 mM l-glutamine, 50 units/ml penicillin and 50 mg/ml streptomycin.

### Cell survival assay for calibration and gene validations

Cells were seeded on matrigel-coated (Corning) 96 well plates, 9,000 cells in 100 μL mTeSR (STEMCELL Technologies) for each well. One plate was seeded for each timepoint. In each plate, 3 wells were seeded for each treatment concentration serving as replicates. 24 h after seeding, the cells were treated with increasing concentrations of Nutlin-3a (Sigma–Aldrich Co.St. Louis, MO, USA). Medium was changed daily for plates until day of measurement (not included). Before each measurement, 100 μL CellTiter-Glo® (Promega, Madison, WI, USA) was added to each well in the plate used for the measurement. The plate was put on a shaker in the dark for 15 min. Finally, the contents of each well were moved to a white 96-well plate and taken for measurement in the BioTek Synergy H1 plate reader. The results of each treated well was normalized to relevant control wells.

### CRISPR screening

Haploid hESC CRISPR-Cas9 library cells were thawed and then grown as described above. When cells reached near confluency, they were harvested, counted and re-seeded on new Matrigel coated plates. 24 h after passaging, control plates were harvested for DNA extraction. In addition, we replaced the medium for the remaining plates with fresh mTeSR containing 14 μM and 18 μM Nutlin-3a (Sigma–Aldrich Co.St. Louis, MO, USA). Cell death and proliferation was monitored with daily replacement of fresh untreated mTeSR medium.

After several days, a fraction of the cells recovered. These cells were treated again with Nutlin. Half of the cells previously treated with 14 μM of Nutlin were treated with 16 μM while the other half continued to be treated with 14 μM. The cells which were treated with 18 μM of Nutlin were treated again with the same concentration.

A fraction of surviving cells that proliferated and reached confluency were harvested for DNA extraction and the rest were reseeded. 24 h after passaging the cells were treated with the relevant concentrations. Once the cells proliferated again, they were given another round of treatment. Finally, when the cells recovered, they were harvested for genome DNA extraction. In conclusion, we had a total of 7 samples; a control, and two timepoints for each of the 3 concentrations.

### DNA extraction

Genomic DNA was extracted using Blood & Cell Culture DNA Midi Kit (Qiagen, Hilden, Germany) in accordance with the manufacturer’s instructions.

### PCR amplification of sgRNAs and high-throughput-sequencing

The sgRNA region, which contains the integrated sgRNA, was amplified using primers with overhang sequenced that are compatible for Nextera DNA library preparation (Illumina, San Diego, CA, USA). The 160 bp product was purified and used to generate a Nextera DNA library by conducting an additional PCR reaction using Nextera adapter primers. The DNA libraries produced from the different samples collected throughout the experiment were sequenced using NextSeq 500 (Illumina).

### Differential expression analysis

Sequencing results were analyzed and merged into a single counts table containing the reads count for the 7 samples. A differential expression (DE) analysis was performed using the edgeR package in R (Robinson et al. 2010). Each of the two timepoints was treated as a replicate consisting of the three different concentrations used in the experiment. Each replicate, along with the control, was transformed into a DGEList and normalized using the trimmed mean of M-values (TMM) method. The dispersion for each replicate was calculated using the estimateDisp function and the glmFit and glmLRT functions were used to fit the sgRNA specific model and conduct the testing between the treatment and control sample respectively. The topTags function was then used to list the top ranked sgRNAs sorted by the p-value.

### Calculating CRISPR score and statistical significance for each gene

The log fold change (logFC), which we used as a CRISPR score for each gene was calculated as the median of the logFC of all the gene’s sgRNAs from the two replicates. In other words, the CRISPR score of each sgRNA was calculated as the ratio of sgRNA abundance between the replicate and control population for each gene. The p-value was calculated using the Kolmogorov–Smirnov test function (ks.test). Genes represented by less than 3 sgRNAs were filtered out. The false discovery rate (FDR) was calculated using the p.adjust function.

To identify genes with meaningful enrichment, we applied a CRISPR Score (CS) threshold of 1.5. This threshold is commonly used in CRISPR screen analyses as it corresponds to a moderate log2 fold change (~ 0.58), balancing sensitivity and specificity. The use of log-transformed data helps normalize variations in sgRNA abundance and reduces the influence of outliers, ensuring more accurate hit selection. A CS of 1.5 highlights genes with biologically significant enrichment while minimizing noise from minor fluctuations. Lowering the threshold would increase sensitivity, potentially including weaker or less consistent hits but at the cost of higher false positives. Conversely, raising the threshold would yield more robust hits but might exclude relevant genes with moderate effects. This choice aligns with established practices in genome-wide CRISPR screens, facilitating comparability with previous studies.

### Protein–protein interaction networks and functional enrichment analysis

Genes were put through a multiple proteins analysis in STRING (Szklarczyk et al. 2021, 2023). Enriched Gene Ontology (Biological Process) and KEGG Pathways gene sets were taken from the Functional Enrichment Analysis function of STRING (Szklarczyk et al. 2023).

### Generation of knockout cell lines

sgRNAs were chosen to target each candidate gene. Each sgRNA was cloned into the lentiCRISPR v2 lentiviral vector (kindly gifted by Feng Zhang, Addgene cat. no. 52961). Lentiviruses were produced by transfecting 293 T cells with sgRNA lentiCRISPR v2, pCMV-VSV-G (kindly gifted by Robert Weinberg, Addgene cat. no. 8454) and psPAX2 (kindly gifted by Didier Trono, Addgene cat. no. 12260) plasmids at a 20:10:15 ratio (11.7 μg total) respectively, mixed with polyethyleneimine ‘Max’ (PEI-Max) (Polysciences) at a 1:2 ratio of DNA to PEI-Max. Transfection medium was replaced with standard hESC medium after 18 h and lentivirus containing supernatant medium was harvested 65 h after transfection. Supernatant was spun down at 3000 rpm for 10 min and filtered using a 0.45 mm cellulose acetate filter (Millipore). Filtered supernatant was aliquoted and stored at − 70 °C.

Haploid hESCs culture were trypsinized using trypsin–EDTA (Biological Industries), centrifuged and resuspended in hESC medium containing 10 μM ROCK inhibitor Y-27632 and 8 mg ml^−1^ polybrene (Sigma). Relevant viruses were added to the cell suspension and cells were seeded on feeder layer MEFs (2 viruses containing two different sgRNAs for each gene). 24 h after transduction, medium was replaced with virus free medium containing puromycin (0.3 mg/ml, Sigma). Antibiotic selection was continued until all control cells died (approximately 7–10 days).

sgRNA sequences:

B3GAT3: 5′-GCTGTACCAGCGCGTAGAGG-3′

B3GALT6: 5′-AGCCCCAGTAGAGGCGGCGG-3′

DPH1: 5′-TGACAGACTCCAGATGGAAG-3′

DNAJC24: 5′-AAAGGATTGGTACAGCATCC-3′

TRIP12*: 5′-**GAAGCGCAAACAGGCCCTGG**−3′; 5′-CAGATGGTGCGATATGGCAG-3′

Both guides were used for mass culture experiment. Guide in bold was used for *TRIP12* KO clone.

### RNA isolation and RNA sequencing

RNA was extracted using RNeasy Mini Kit (Qiagen, Hilden, Germany). RNA sequencing libraries were generated using TruSeq RNA Library Prep Kit (Illumina) and sequenced using Illumina NextSeq 500 with 75 bp single-end reads. The reads were mapped to GRCh38 and aligned to GRCm38 using the STAR package (Dobin et al. 2013). Genes that were aligned to GRCm38 were filtered out using XenofilteR in R (Kluin et al. 2018). edgeR was used to establish count tables and calculate statistical significance on filtered results (Robinson et al. 2010).

### Real-time qPCR

RNA was reverse-transcribed into first-strand complementary DNA using qScript cDNA Synthesis Kit (Quantabio, Beverly, MA, USA) according to the manufacturer’s instructions. RT qPCR was performed using PerfeCTa SYBR Green FastMix (Quantabio, Beverly, MA, USA) according to manufacturer’s instructions using 7300 Real-Time PCR System (Applied Biosystems, France). The expression of each target gene was normalized to GAPDH.

Primer Sequences:

GAPDH forward: 5′-AGCCACATCGCTCAGACACC-3′, GAPDH reverse: 5′-GTACTCAGCGCCAGCATCG-3′;

*PMAIP1* forward: 5′-CCTACTGTGAAGGGAGATGA −3′, PMAIP1 reverse: 5′-GCTGAGTTGGCACTGAAA-3′;

p21 forward: 5′-GGAAGACCATGTGGACCTGT −3′, p21 reverse: 5′-GGCGTTTGGAGTGGTAGAAA-3′

### Western blot

Cells lysis was done using 2XSDS + DTT (125 mM Tris, 20% Glycerol, 4% SDS, DTT, pH 6.8) lysis buffer, with incubation at 100 °C for 15 min. Lysed samples were stored at − 20 °C. Before running, cells were incubated at 100 °C for 5 min. Samples were put through electrophoresis on 11% SDS polyacrylamide gel. Wet transfer to nitrocellulose membrane was done at room temperature for 90 min. Blocking was done using TBS-T buffer (0.15 M NaCl, 0.05 M Tris hydroxymethyl methylamine and 0.1% Tween-20, pH 7.4–7.6) containing 5% nonfat milk (BD) for 30 min at room temperature. Membranes were then washed and incubated at 4 °C overnight with primary antibodies: anti-p53 sc-126 (Santa Cruz California, USA), anti-GAPDH ab8245 (Abcam, Cambridge, MA, USA) and anti-USP7 A300-033A (Bethyl Laboratories, Montgomery, TX, USA). Cells were then washed with TBS-T buffer and incubated with secondary antibody for 2 h at room temperature. The membrane was then washed three times with TBS-T for 10 min each and incubated with secondary antibody for 1 h at room temperature. Chemiluminescence visualization was done using ECL reagent kit (Biogate, Ness Ziona, Israel) in ChemiDoc gel imaging system (Bio-Rad, Hercules, CA, USA). Protein quantification was done using the Image Lab Software (Bio-Rad, Hercules, CA, USA).

## Results

### Induction of elevated p53 levels using Nutlin-3a

**Fig. 1:**
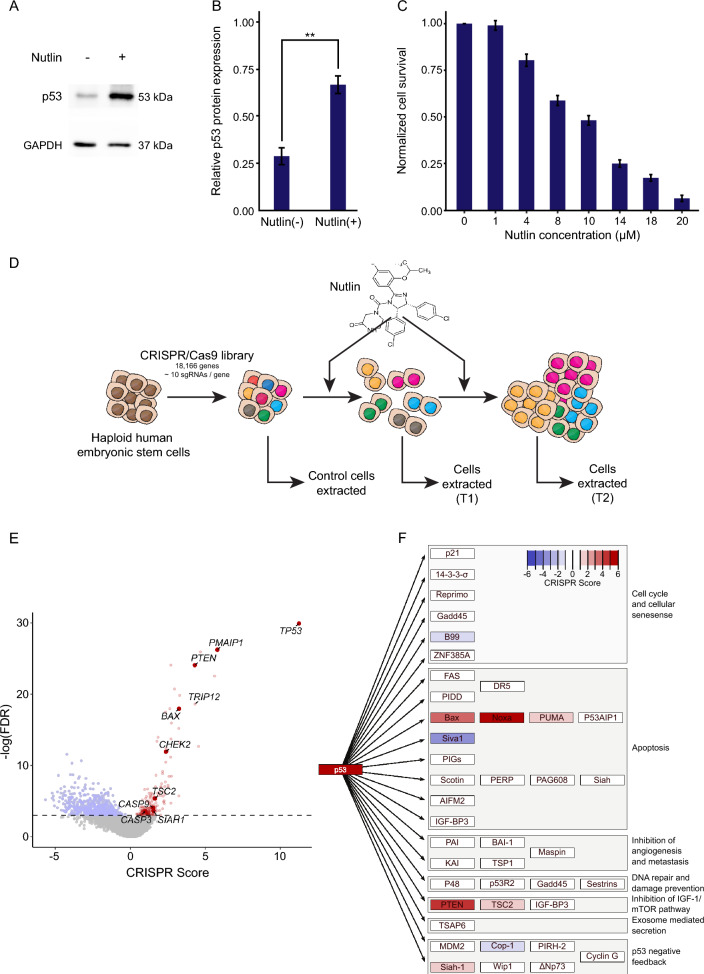
Genome-wide CRISPR library treated with Nutlin to study genes involved in p53-mediated apoptosis. **A** Western blot analysis of changes in p53 protein levels in WT hESCs following treatment with 4 µM Nutlin for 6 h. **B** Quantification of western blot results of p53 levels relative to GAPDH. The graph represents the average of 3 biological repeats with each biological repeat having two technical repeats. Asterisks represent p-value of a two-tailed unpaired t-test between treated and untreated cells. (**p-value < 0.01). Error bars represent standard error of the mean. **C** Cell survival analysis of WT hESCs 72 h after treatment with Nutlin in the indicated concentrations, normalized to untreated cells. The graph represents the average of 3 biological repeats. Error bars represent standard error of the mean. **D** Schematic illustration of experimental overview. Nutlin chemical structure is shown in scheme. **E** Volcano plot representing -log (FDR) and CRISPR Score of all genes in the screen. Dashed line represents FDR = 0.05. Labelled points are p53 pathway genes from KEGG that are enriched and statistically significant (FDR < 0.05), as well as TRIP12, which is a gene of interest in this article. **F** Scheme summarizing target proteins of p53 clustered in functional groups (adapted from the KEGG database). Colors represent CRISPR Score of genes that are statistically significant (p-value < 0.05)

Our aim is to identify, in a genomic scale, genes involved in p53-mediated apoptosis. We have thus tried to increase p53 levels, inducing apoptosis, and then identify genes that their loss-of-function will rescue the cells from p53-induced apoptosis. The small molecule Nutlin is an MDM2 antagonist that leads to the stabilization of the p53 protein, resulting in an increase in its levels (Vassilev et al. 2004). To verify that Nutlin treatment leads to an increase in p53 protein levels in the haploid hESC line h-pES10, we performed a western blot analysis on Nutlin-treated cells. An approximately two-fold increase in p53 levels was observed in response to Nutlin treatment (Fig. 1A, B). To choose the appropriate concentrations of Nutlin for our genome-wide screen, we conducted a survival assay using different doses of Nutlin treatment. A 70–85% decrease in cell survival was observed 72 h after treatment with 14–18 µM of Nutlin, compared to the control cells (Fig. 1C). Moreover, after approximately 96 h, the cells were able to recover and proliferate (Supplementary Fig. 1). Based on these results, we chose 3 different concentrations (14, 16 and 18 µM) of Nutlin to be used in our screen.

To further clarify the nature of cell survival following Nutlin treatment, it is important to note that the observed recovery and proliferation (Fig. 1C, Supplementary Fig. 1) primarily reflect the behavior of the 15–30% of cells that initially survived the treatment. These survivors include cells that either underwent transient growth arrest or harbored sgRNA-induced genetic alterations that conferred resistance to p53-mediated apoptosis. While a substantial proportion of cells succumbed to apoptosis, the surviving fraction retained the capacity to re-enter the cell cycle and proliferate once the selective pressure was reduced. This strategy ensured that a sufficient number of viable cells were available for downstream analyses, including sgRNA abundance sequencing.

### CRISPR screening to identify genes involved in p53-mediated apoptosis

For the genome-wide LoF screening, we have used our previously published mutation library in haploid human embryonic stem cells utilizing over 180,000 gRNAs, targeting almost all protein-coding genes (> 18,000 genes) (Yilmaz et al. 2018). The library of cells was treated with 14–18 µM Nutlin, while untreated cells were extracted as a control (Fig. 1D). After massive cell death, some cells survived, recovered and proliferated again. A portion of them was extracted for the first timepoint while the rest were passaged on to new plates and grown for 24 h before being treated again with Nutlin. After the recovery of the cells, they were extracted for sequencing for the second timepoint. Cells were extracted at two timepoints for each concentration in addition to the control samples which were harvested 24 h after the cells were seeded (Fig. 1D). Genomic DNA was extracted from each sample, and DNA regions containing the sgRNA were amplified and sent for sequencing.

Sequencing results were analyzed and the abundance of each sgRNA was calculated for each sample. To investigate significant changes in sgRNA representation, we calculated a CS for each gene as the ratio of its sgRNAs’ abundance between the control and treated samples. 736 genes have a statistically significant CS (FDR < 0.05), 178 of which were enriched with a CS over zero (Fig. 1E, Supplementary Table 1). Unsurprisingly, TP53 was the most enriched gene in our screen, with a CS of 11.25.

### Identification of genes and pathways involved in p53-mediated apoptosis

Since this screen was centered around the effect of p53 upregulation, we checked the screen results for p53 KEGG pathway genes. Among the different p53 pathways, the Bax/Noxa (*BBC3*)/PUMA (*PMAIP1*) and the PTEN/TSC2 pathways are the two most enriched, indicating that they are the most prominent downstream pathways of p53 to regulate apoptosis (Fig. 1E, F and Supplementary Table 1). In addition, *TRIP12*, which is not a known canonical p53 pathway gene, was observed to be highly enriched in the screen (Fig. 1E).

**Fig. 2:**
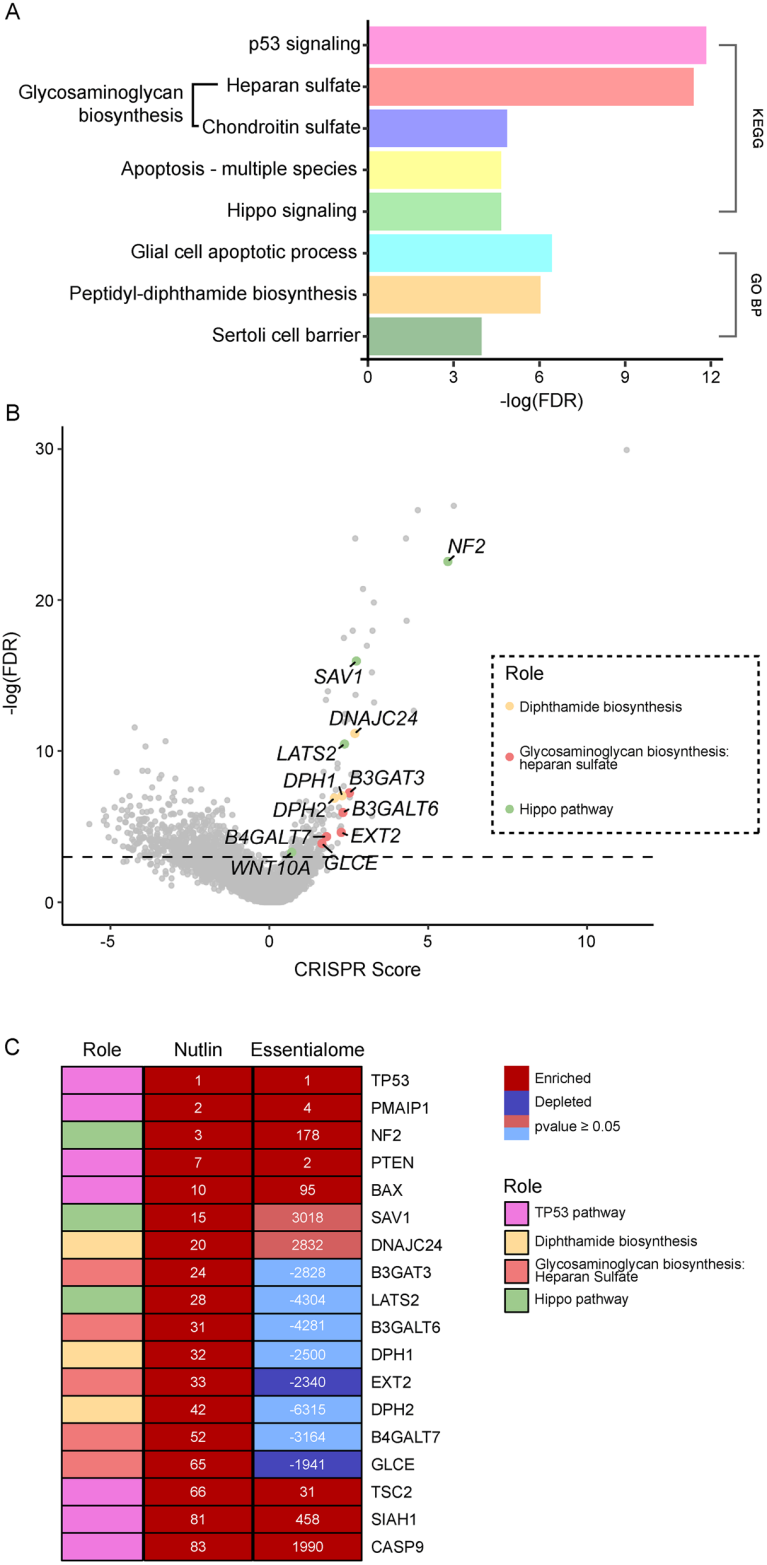
Gene set enrichment analysis to examine genes identified in screen. **A.** Bar plot showing -log (FDR) of enriched gene sets from KEGG and GO Biological Process analysis. **B.** Screen volcano plot representing -log (FDR) and CRISPR Score of all genes in screen. Enriched and statistically significant genes (FDR < 0.05) belonging to pathways of interest are colored and labelled. Dashed line represents FDR = 0.05. **C.** Table comparing the rank of genes in that are part of the enriched gene sets of interest in Nutlin and untreated (control) screens

**Fig. 3:**
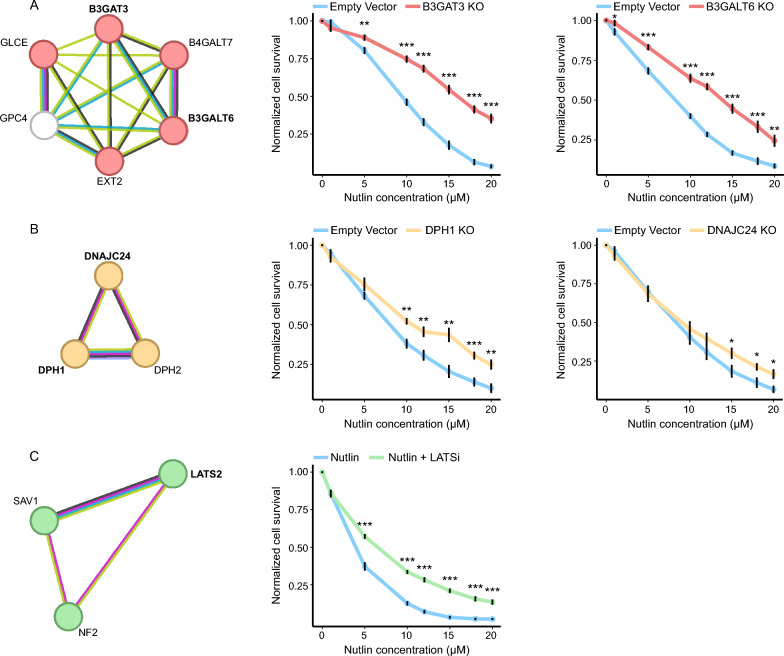
Validation of genes belonging pathways of interest. **A** (left) STRING analysis of heparan sulfate glycosaminoglycan biosynthesis genes that are statistically significant (FDR < 0.05) and have a CRISPR Score > 1.5. Genes that were knocked out for validation are highlighted in bold**.** Normalized cell survival as a function of Nutlin concentration for *B3GAT3* (middle) and *B3GALT6* (right) KO cell lines compared to empty vector control cells. The graph represents the average of 6 biological repeats in 2 independent experiments. Asterisks represent p-value of two-tailed paired t-test between each data point in knockout cells and WT cells. (*p-value < 0.05, **p-value < 0.01 and ***p-value < 0.001). Error bars represent standard error of the mean. **B** (left) STRING analysis of diphthamide biosynthesis genes that are statistically significant (FDR < 0.05) and have a CRISPR Score > 1.5. Asterisks mark genes that were knocked out for validation. Normalized cell survival as a function of Nutlin concentration for *DPH1* (middle) and *DNAJC24* (right) KO cell lines compared to empty vector control cells. The graph represents the average of 6 biological repeats in 2 independent experiments. Asterisks represent p-value of two-tailed paired t-test between each data point in knockout cells and WT cells. (*p-value < 0.05, **p-value < 0.01 and ***p-value < 0.001). Error bars represent standard error of the mean. **C** (left) STRING analysis of Hippo pathway genes that are statistically significant (FDR < 0.05) and have a CRISPR Score > 1.5. Asterisks mark target gene (left). Normalized cell survival as a function of Nutlin concentration for cells treated with the LATSi compared to control cells (not treated with LATSi) (right). The graph represents the average of 6 biological repeats in 2 independent experiments. Asterisks represent p-value of two-tailed paired t-test between each data point in knockout cells and WT cells. (***p-value < 0.001). Error bars represent standard error of the mean

Functional enrichment and protein–protein interaction networks analysis was conducted on statistically significant genes (FDR < 0.05) with a CS that is greater than 1.5 (76 genes), using STRING (Fig. 2A and Supplementary Table 2). As expected, p53 signaling is the most enriched KEGG gene set in the functional enrichment analysis. Interestingly, KEGG gene sets involved in the biosynthesis of glycosaminoglycans with HS, chondroitin sulfate, and dermatan sulfate were enriched in our screen (Fig. 2). In addition, Hippo signaling pathway genes, which are known to coordinate cell proliferation, differentiation and cell death (Zhao et al. 2011; Pan 2010), were also among the highest enriched gene sets in our screen (Fig. 2). Moreover, a unique group of genes involved in the diphthamide biosynthesis pathway, which is a post-translation modification that occurs on eEF2 and required for accurate translation (Zhu et al. 2011; Liu et al. 2004, 2012; Thakur et al. 2012; Hawer et al. 2020, 2018), were enriched in our screen (Fig. 2). Furthermore, clear clusters can be seen in the protein–protein interaction network obtained from STRING for these gene networks (Fig. 3A–C; left STRING analysis and Supplementary Fig. 2).

Genes belonging to the groups mentioned above are among the most prominent genes that are significantly enriched in our screen (Fig. 2B, C). To see if these observations are due to these genes’ link to p53-mediated apoptosis or cell cycle arrest and not due to a general essentiality of these genes, we compared their rank in our screen to their rank in our previous essentialome screen (hereby referred to as the control screen) (Yilmaz et al. 2018) (Fig. 2C). Main p53 pathway genes such as *PMAIP1* and *PTEN* are highly ranked in both screens, an indication that the essentiality of these genes, which was observed in untreated cells, is linked to their interaction with p53.

### Genetic validation of B3GAT3, B3GALT6, DPH1 and DNAJC24 as players in p53 mediated apoptosis

HS glycosaminoglycan biosynthesis genes showed a significant survival advantage in cells treated with Nutlin. These genes were significantly enriched in this screen having high ranks among the significantly enriched genes while the same genes were depleted in the control screen (Fig. 2B, C).

Diphthamide biosynthesis were also among the highly ranked genes in our screen. *DPH1* and *DPH2*, which work closely together in diphthamide biosynthesis (Liu et al. 2004; Schaffrath et al. 2014), are significantly enriched in our screen while they were not statistically significant and depleted in the control screen (Fig. 2B,C). In addition, *DNAJC24*, another gene involved in this pathway, is among the highly ranked enriched genes in this screen while having a low rank and statistically insignificant CS in the control screen (Fig. 2B, C).

To verify the results obtained in this screen, we generated KO h-pES10 cells for each of: *B3GAT3* and *B3GALT6* (glycosaminoglycan synthesis), *DPH1* and *DNAJC24* (diphthamide biosynthesis). In addition, we infected wild type h-pES10 cells with an empty vector that does not contain any guide to be used as a control. To validate our results, we conducted viability assays where we exposed KO and empty vector cells to different concentrations of Nutlin and compared their sensitivity. These 4 gene KOs made the cells less sensitive to Nutlin, further validating that the disruption of these pathways in a p53 upregulation background gives the cells a growth advantage compared to WT cells (Fig. 3A, B). These results indicate that the production of HS glycosaminoglycans and the diphthamide modification are components of p53’s mechanism of action.

### Chemical inhibition of the Hippo pathway helps the cells evade p53 mediated apoptosis

Genes belonging to the Hippo pathway are also among the significantly enriched genes with high rankings in our screen. While *NF2* was also significantly enriched in our control screen, it has a more prominent ranking in our screen, ranking 3rd in comparison with 178th in the control screen (Fig. 2C). This indicates that the essentiality of this gene is increased after applying the selective pressure of Nutlin. *SAV1* and *LATS2*, both of which are central components of the Hippo pathway, are among the top enriched genes in our screen while scoring a very low ranking in the screen with no significant p-value (Fig. 2C).

Therefore, we aimed to validate the effect of Hippo pathway inhibition on the cell’s sensitivity to Nutlin. We conducted a survival assay using the Hippo pathway inhibitor TRULi (hereby referred to as LATSi), a potent and ATP-competitive inhibitor of Lats1 and Lats2 kinases (Kastan et al. 2021). WT h-pES10 cells were treated with different doses of Nutlin in addition to LATSi and compared to cells that were treated only with Nutlin. Results show that the addition of LATSi decreased the sensitivity of the cells to Nutlin (Fig. 3C). This result highlights the cooperation of the p53 and Hippo in restricting cell growth and apoptosis.

### Investigating the mechanism of decreased sensitivity to p53 upregulation in TRIP12 KO cells

**Fig. 4:**
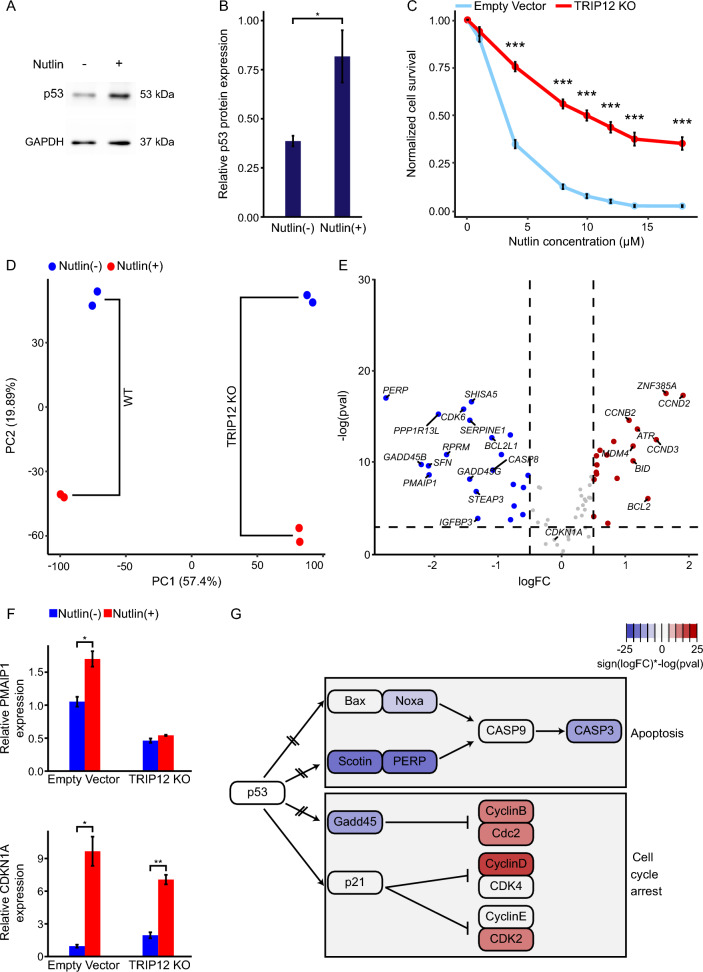
Validation and investigation of TRIP12. **A** Western blot analysis of change in p53 protein levels in *TRIP12* KO clone cells following treatment with 4 µM Nutlin for 6 h. **B** Quantification of western blot results of p53 relative to GAPDH. The graph represents the average of 3 biological repeats with each biological repeat having two technical repeats. Asterisks represent p-value of two-tailed unpaired t-test between treated and untreated cells. (*p-value < 0.05). Error bars represent standard error of the mean. **C** Normalized cell survival as a function of Nutlin concentration for *TRIP12* KO clone compared to empty vector control cells. The graph represents the average of 6 biological repeats in 2 independent experiments. Asterisks represent p-value of t-test between each data point in knockout cells and WT cells. (***p-value < 0.001). Error bars represent standard error of the mean. **D** Principal component analysis plot demonstrating separation of samples from two biological repeats of RNA-Seq of *TRIP12* KO clone and empty vector cells that were either untreated or treated with Nutlin. **E** Volcano plot demonstrating -log (p-value) and logFC of p53 KEGG pathway genes in differential expression analysis of treated samples (6 µM, 24 h) of *TRIP12* KO clone and empty vector. The analysis was performed with three biological replicates (n = 3) per condition. Horizontal dashed line indicates p-value = 0.05. Vertical dashed lines mark logFC of −0.5 and 0.5. **F** Relative expression analysis (qRT-PCR) for *PMAIP1* (Noxa) on left and *CDKN1A* (p21) on right in Nutlin treated (6 µM, 24 h) and untreated samples of *TRIP12* KO clone and empty vector cells. Each bar represents replicates from 3 independent experiments. Asterisks represent p-value of two-tailed unpaired t-test between treated and untreated cells. (*p-value < 0.05, **p-value < 0.01). Error bars represent standard error of the mean. **G** Schematic summarizing results observed in differential expression highlighting p53 pathway genes that showed notable difference in expression between *TRIP12* KO clone and empty vector cells after Nutlin treatment. Color scale represents the sign of the logFC multiplied by the log of the p-value for each gene

*TRIP12* is an E3 ubiquitin ligase that regulates various genes that are involved in the DNA damage response. It is significantly enriched in this screen having the 6th highest CS among the genes with an FDR < 0.05 while having a negative CS (FDR > 0.05) in our control screen. To investigate this gene’s role in p53 mediated apoptosis, we created a *TRIP12* KO clone to examine whether this knockout affects accumulation of p53 protein induced by Nutlin or affects the transcriptional activation of downstream genes by p53 (Supplementary Fig. 3A). In parallel, we created an empty vector clone to be used as a control. Similar to control cells (Fig. 1A, B), results show an increase in p53 protein levels ruling out that the decrease in sensitivity to Nutlin is caused by a drastic change in p53 protein levels (Fig. 4A, B). To verify the result that was obtained in our screen, we conducted a cell viability assay on *TRIP12* knockout clone. These cells clearly showed lower sensitivity to Nutlin compared to empty vector (control) cells (Fig. 4C). Similar results were observed for both experiments in mass culture *TRIP12* KO cells (Supplementary Fig. 3B).

Interestingly, given that TRIP12 is known to target USP7 for proteasomal degradation (Liu et al. 2016), we examined whether USP7 protein level was affected in our TRIP12 KO cells under both basal and Nutlin-treated conditions. Western blot analysis revealed no significant difference in USP7 protein levels between TRIP12 KO and control cells, regardless of Nutlin treatment (Supplementary Fig. 3D,E). This indicates that, in our system, the absence of TRIP12 does not impact USP7 stability, even under conditions that activate the p53 pathway.

p53 is a transcription factor and therefore treatment with Nutlin, which augments its levels, is expected to change gene expression in cells. Next, we wanted to check if *TRIP12* KO shows any changes in the transcription of the p53 pathway genes in comparison to WT cells in response to Nutlin treatment. We conducted RNA-sequencing to treated and untreated samples of *TRIP12* KO and empty vector clone cells. Using clones for this experiment was important in order to minimize sequencing noise that may be obtained in mass culture KO cells.

The PCA plot demonstrates a clear separation between control and *TRIP12* KO samples and between the treated and untreated samples (Fig. 4D), indicating a difference in gene expression patterns in the different types of cells and conditions. To make sure that TRIP12 transcription is not affected by p53 upregulation, we conducted differential expression analysis of treated and untreated samples of empty vector control cells. No significant change in TRIP12 expression was observed in response to Nutlin treatment (Supplementary Fig. 3C).

Differential expression analysis between the treated samples of control and *TRIP12* KO cells shows a difference in the expression of some canonical p53 pathway genes (Fig. 4E). Noxa (PMAIP1), which had a high CS in the Nutlin screen (Fig. 1E, F), is downregulated in TRIP12 KO cells compared to control (Fig. 4E and Supplementary Table 3). This was also verified using RT-PCR where PMAIP1 levels were upregulated in empty vector cells upon treatment with Nutlin, while no significant upregulation was observed in TRIP12 KO cells (Fig. 4F). To make sure that this effect was specific for PMAIP1 and not a result of a general interference with p53 function as a transcription factor, we checked CDKN1A expression levels in the same conditions. We see a similar pattern of upregulation of CDKN1A after Nutlin treatment in TRIP12 KO and control cells indicating that the deterred upregulation of *PMAIP1* in *TRIP12* KO cells is specific to this gene (Fig. 4F).

Interestingly, apart from Noxa, other p53 downstream genes were downregulated in *TRIP12* KO cells. The *SHISA5* (Scotin) and PERP genes, both of which are in the same downstream pathway and are important for the tumor suppressor activity of p53 (Ihrie et al. 2003; Bourdon et al. 2002), are downregulated in *TRIP12* KO cells. In addition, *GADD45B* and *GADD45G*, two members of the *GADD45* gene family, which promotes cell cycle arrest in response to DNA damage (Wang et al. 1999; Jin et al. 2002), are also downregulated in *TRIP12* KO cells (Fig. 4E and Supplementary Table 3). Based on these findings, we propose a model, where reduced sensitivity to p53 accumulation in *TRIP12* KO cells is through interference with the regulation of several p53 downstream genes involved in apoptosis and cell cycle arrest (Fig. 4G).

## Discussion

The *TP53* gene is the most studied gene in cancer biology, with over 100,000 PubMed citations. This is mainly due to its importance in the suppression of tumors and the wide variety of effects it may have when mutated. TP53 gene alterations through loss of function, dominant negative effects, or gain of function greatly increase the probability of cancer.

The extensive investigation of p53 has revealed its role in various gene networks. However, it is clear that the complexity of this gene may still hide unknown or overlooked interactions and modes of action. Finding new roles of p53 may help in developing cancer treatment and in the assessment of the appropriate chemotherapies to be used to treat patients.

In this study, we conducted a screen using Nutlin to elevate p53 levels in our CRISPR-Cas9 mutant library hESCs. Since we introduced a remarkable selective pressure on the cells by inducing p53-mediated mass cell death and growth restriction, we were mainly interested in the genes that are enriched and statistically significant.

The use of haploid hESCs in this study provided a powerful and efficient platform for functional genomic screening. Haploid hESCs offer unique advantages for CRISPR-based screens by allowing the generation of gene knockouts without the need to target both alleles, significantly simplifying genetic manipulation. Notably, these cells undergo endoreduplication during extended culture, resulting in diploid cells carrying homozygous mutations, a process previously described (Yilmaz et al. 2018; Sagi et al. 2016a, 2016b). As a result of this natural diploidization, a significant portion of cells in the culture are diploid by the end of the experiment, effectively mitigating concerns about haploid-specific artifacts. Importantly, haploid hESCs maintain key biological properties comparable to diploid hESCs, including similar proliferation rates, differentiation capacity, transcriptomic profiles, and epigenetic landscapes (Yilmaz et al. 2018; Sagi et al. 2016a). These attributes not only validate the use of haploid hESCs in large-scale functional screens but also highlight their utility in elucidating complex gene networks, such as the p53 pathway.

Similarly to the control screen previously conducted in our lab (Yilmaz et al. 2018), TP53 is the most enriched and highly significant gene in our screen, highlighting the importance and robustness of this gene’s role in restricting proliferation. Moreover, as is the case with the control screen, several genes involved in the canonical p53 pathways are enriched further strengthening the integral role of the p53 network in controlling the proliferation of stem cell culture. Among all the canonical pathways of p53 listed in KEGG, the Bax-Noxa-PUMA and PTEN axis seem to be the most prominent downstream p53 pathways in growth restriction in hESCs. While Bax, Noxa, and PTEN were also significantly enriched in the control screen, PUMA was not statistically enriched, indicating that its growth inhibition role is strictly p53-dependent.

In addition to the canonical p53 pathway genes that are enriched in this screen, there are other related genes, *USP28* and *TP53BP1*, which are significantly enriched (Supplementary Table 1). This further confirms former studies showing that these two genes interact together with p53 leading to its stabilization and activation (Lambrus et al. 2016; Meitinger et al. 2016). Moreover, *USP28*, but not *TP53BP1*, is significantly enriched in the control screen. *USP28* and *TP53BP1* are also significantly enriched in genome-wide screenings for resistance to anti-cancer drugs (in 6 or 9 out of the 10 anticancer drug screens, for *TP53BP1* or *USP28*, respectively (Segal et al. 2023)). These results highlight the importance of these genes suggesting that they may serve as potential targets for cancer therapy.

Functional enrichment analysis of the top enriched genes showed three gene sets that were enriched in our screen (Fig. 2A). Among these groups were the genes involved in HS glycosaminoglycan biosynthesis. HS glycosaminoglycans are complex macromolecules that composed of a protein core that is covalently attached to linear sulfated polysaccharide chains (Gallagher et al. 1986). These glycosaminoglycans have various functions, including acting as scaffolds for protein–protein interactions, modulating extracellular ligand gradients, and influencing proliferation (Matsuo and Kimura-Yoshida 2014; Karamanos et al. 2018; Li et al. 2008; Vlodavsky et al. 1999). Moreover, HS glycosaminoglycans play crucial roles in regulating stem cell function and cancer progression by regulating important signalling pathways and interacting with growth factors (Vitale et al. 2019; Grunert et al. 2008). Notably, Heparanase, which degrades HS (Hulett et al. 1999; Kussie et al. 1999) and is downregulated by p53 (Baraz et al. 2006), is highly expressed in tumor cells (Vlodavsky et al. 1999; Kosir et al. 1999). Therefore, the negative regulation of this protein by p53, which leads to the stabilization of HS is crucial for maintaining a healthy cell. In our study, we showed that when we target central genes involved in the biosynthesis of HS glycosaminoglycans, the cells become resistant to p53-mediated growth inhibition, suggesting that the regulation of HS is another crucial part of p53 biology. However, while our data indicate a connection between HS glycosaminoglycan biosynthesis and p53-mediated pathways, it remains possible that this interaction is indirect, and further studies are required to delineate the precise nature of this relationship.

Genes involved in the biosynthesis of diphthamide were also significantly enriched in our screen. Diphthamide is a unique posttranslational modification found only on the histidine residue of eEF2 and is conserved in archaea and eukaryotes (Schaffrath et al. 2014). This modification has been extensively studied because it is targeted by the diphtheria toxin which is a potent exotoxin produced by *Corynebacterium diphtheriae* leading to the ADP-ribosylation of the eEF2 protein at the diphthamide residue causing diphtheria and upper respiratory tract infection (Schaffrath et al. 2014). It has been shown that cells containing a knockout of *DPH1*, *DPH2* and *DNAJC24* produce eEF2 that does not contain the diphthamide modification (Stahl et al. 2015; Webb et al. 2008). While our findings indicate that knockout of diphthamide biosynthesis genes (such as DPH1 and DNAJC24) results in reduced sensitivity to Nutlin-mediated p53 upregulation, we acknowledge that the mechanistic link between diphthamide-modified eEF2 and p53 function remains speculative.

p53 is known to interact directly with eEF2 (Yin et al. 2003; Argüelles et al. 2013). In neurons, p53 binds eEF2 and increases its localization to the nucleus (Argüelles et al. 2013). Additionally, p53 covalently links with 5.8S rRNA (Fontoura et al. 1992, 1997). These interactions with key components of the translational machinery highlight p53’s role in regulating translation in response to cellular stress. Our hypothesis suggests that diphthamide-modified eEF2 could influence the translation of specific p53 target genes, yet direct experimental evidence supporting this connection is currently lacking. Future studies focusing on the translational regulation of p53 target genes in the context of diphthamide deficiency could provide deeper mechanistic insights.

The Hippo signaling pathway is involved in organ size regulation, cell proliferation and apoptosis (Zhao et al. 2011; Pan 2010; Furth and Aylon 2017; Varelas 2014). Interestingly, members from the different levels of this pathway are enriched in our screen. *NF2*, which is among the most highly enriched genes in our screen, promotes *LATS1/2*-dependent phosphorylation of YAP (Cockburn et al. 2013). Respectively, *LATS2*, is also highly enriched gene in our screen. *LATS1/2* are an integral part of the Hippo pathway, responsible for phosphorylating YAP and TAZ, which prevents them from translocating into the nucleus and interacting with TEAD (Pan 2010). When the Hippo pathway is not activated, YAP/TAZ are localized into the nucleus where they bind TEAD and activate genes that are responsible for proliferation (Pan 2010). In addition, *SAV1*, which works upstream to LATS1/2, is also highly enriched in our screen. *SAV1* functions as a scaffold for MAP4Ks and MST1/2 which together are responsible for phosphorylating LATS1/2 (Yin et al. 2013).

The Hippo pathway cooperates with p53 at various levels in inducing senescence and apoptosis (Raj and Bam 2019). For example, LATS2, which is enriched in our screen, can activate p53 and is transcriptionally activated by p53 (Aylon et al. 2010). Additionally, p53 and YAP form a positive feedback loop by upregulating each other’s expression (Bai et al. 2013).

Our results showed that inhibiting the Hippo pathway makes the cells less sensitive to Nutlin, marking the importance of the cooperation of the Hippo and p53 pathways. While our findings highlight the possible involvement of LATS1/2, we did not directly test whether inactivation or knockdown of LATS1/2 affects the transcriptional activity of p53.

*TRIP12* is among the most highly enriched genes in this screen. This E3 ubiquitin ligase is involved in ubiquitination of various genes involved in DNA repair (Gudjonsson et al. 2012). While no direct interaction between *TRIP12* and p53 has been reported, *TRIP12* interacts and ubiquitinates genes known to be implicated with p53 (Liu et al. 2016; Chen et al. 2010). For example, *TRIP12* has been shown to be involved in the ubiquitination of USP7 which can stabilize both p53 and MDM2 (Liu et al. 2016; Li et al. 2002, 2004). Furthermore, *TRIP12* has been found to mediate the ubiquitination of isoform p19ARF of *CDKN2A* which stabilizes p53 through the inhibition of its MDM2 mediated degradation (Chen et al. 2010; Tao and Levine 1999).

In our study, we used Nutlin to stabilize p53, effectively bypassing mechanisms that primarily target the MDM2-p53 axis. Therefore, these known TRIP12 interactions alone cannot fully explain the observed decreased sensitivity to p53 upregulation in TRIP12 KO cells. To further explore a potential role for USP7, we performed western blot analysis to assess USP7 protein levels in both empty vector and TRIP12 KO cells, with and without Nutlin treatment. The results revealed no significant differences in USP7 protein levels across conditions. This suggests that altered USP7 abundance is unlikely to account for the decreased Nutlin sensitivity in these cells. However, we recognize that USP7 activity could still be modulated independently of its protein levels, such as through changes in post-translational modifications or ubiquitination status, which may influence downstream effects on p53. Future studies, including proteomic analyses and assays directly measuring USP7 activity, are needed to further investigate this possibility.

Our data confirm that TRIP12 transcription remains unchanged following Nutlin treatment in control cells, indicating that it is neither a direct nor an indirect transcriptional target of p53 (Supplementary Fig. 3C). However, when comparing control and TRIP12 cells treated with Nutlin, we observed a significant difference in the transcription of several p53 canonical target genes, including Noxa, which is the second most enriched gene in our CRISPR screen. Therefore, this altered regulation of these downstream genes in *TRIP12* KO cells may be the driver behind the resistance of these cells to p53 upregulation. Future studies, including proteomic analyses of TRIP12 and its interaction network, are warranted to elucidate the post-translational modifications and downstream targets that mediate TRIP12’s impact on p53 signaling.

Overall, we have conducted a genome-wide CRISPR screen using our robust system of haploid cells and abundant guides for each gene. We have shed light on 3 groups of genes of great importance to p53-mediated apoptosis and growth inhibition. In addition, we have shown that *TRIP12* is important for p53-meidtaed apoptosis and growth inhibition, probably through p53's function as a transcription factor. Our findings contribute valuable insights into the p53 pathway and suggest novel therapeutic targets for the treatment of cancer.

## Conclusion

In conclusion, our study has revealed novel insights into the mechanisms underlying the p53-pathway. Through a comprehensive CRISPR-Cas9 screen utilizing Nutlin-3a, we identified key pathways that modulate resistance to p53 upregulation, including heparan sulfate glycosaminoglycan biosynthesis, diphthamide biosynthesis, and the Hippo pathway. Notably, TRIP12 emerged as a critical regulator required for p53-dependent transcription of pro-apoptotic genes. These findings not only deepen our understanding of p53's multifaceted role in tumor suppression but also offer promising avenues for developing targeted therapies aimed at enhancing p53-mediated responses in cancer cells.

## Supplementary Information

Additional file 1.

Additional file 2.

Additional file 3.

Additional file 4.

Additional file 5.

Additional file 6.

Additional file 7.

## Data Availability

No datasets were generated or analysed during the current study.

## Abbreviations

KO: Knock out
WT: Wild type
eEF2: Eukaryotic elongation factor 2 (eEF2)
LoF: Loss of function
hESC: Human embryonic stem cells
HS: Heparan sulfate
DE: Differential expression
TMM: Trimmed mean of M-values
logFC: Log fold change
ks.test: Kolmogorov–Smirnov test function
FDR: False discovery rate
CS: CRISPR score
