# Supplementary figures and images for: Genome-wide screening in human embryonic stem cells identifies genes and pathways involved in the p53 pathway
Source: Mol Med. 2025 Mar 13;31:97. doi: 10.1186/s10020-025-01141-5 (PMC11907909; doi:10.1186/s10020-025-01141-5)

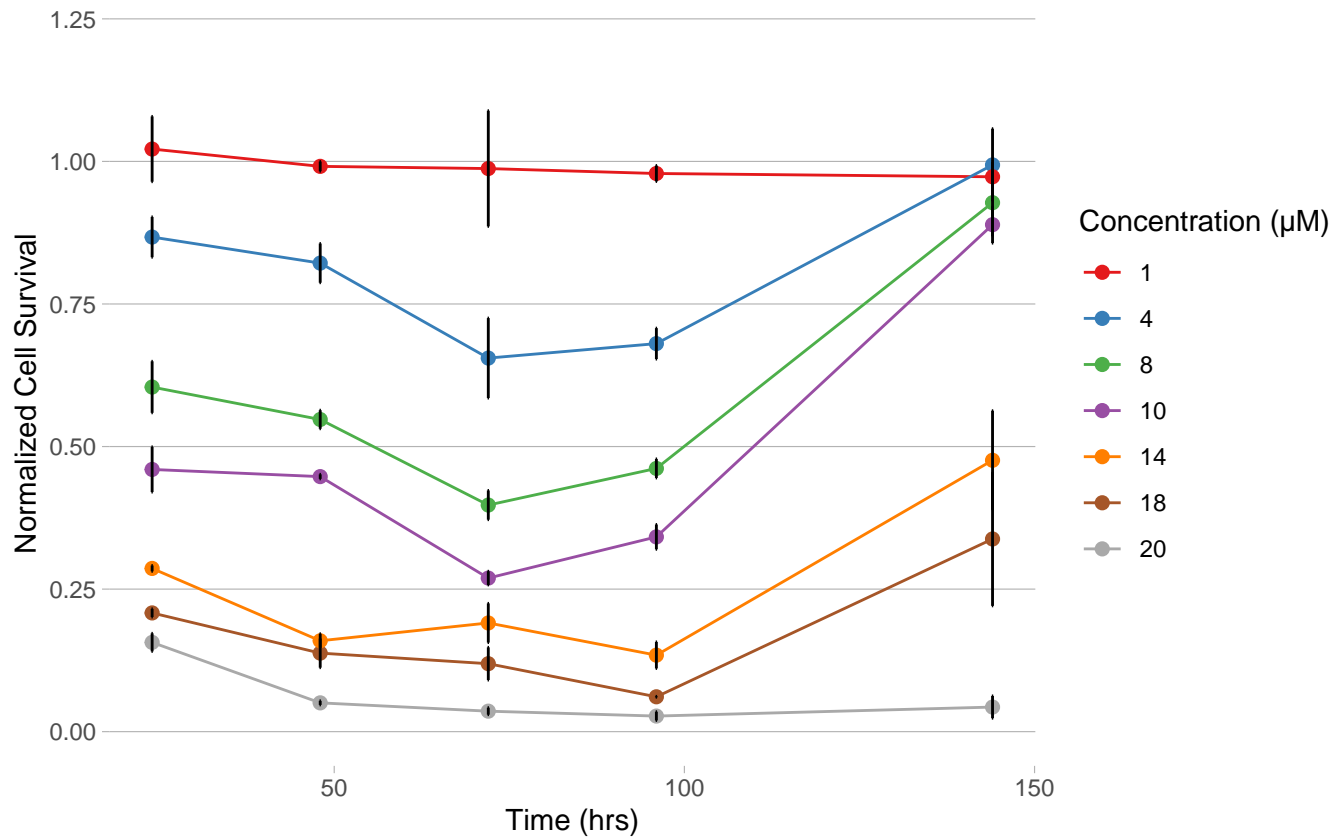

Supplement: Supplementary file 4 — Additional file 4. [file 10020_2025_1141_MOESM4_ESM.pdf]

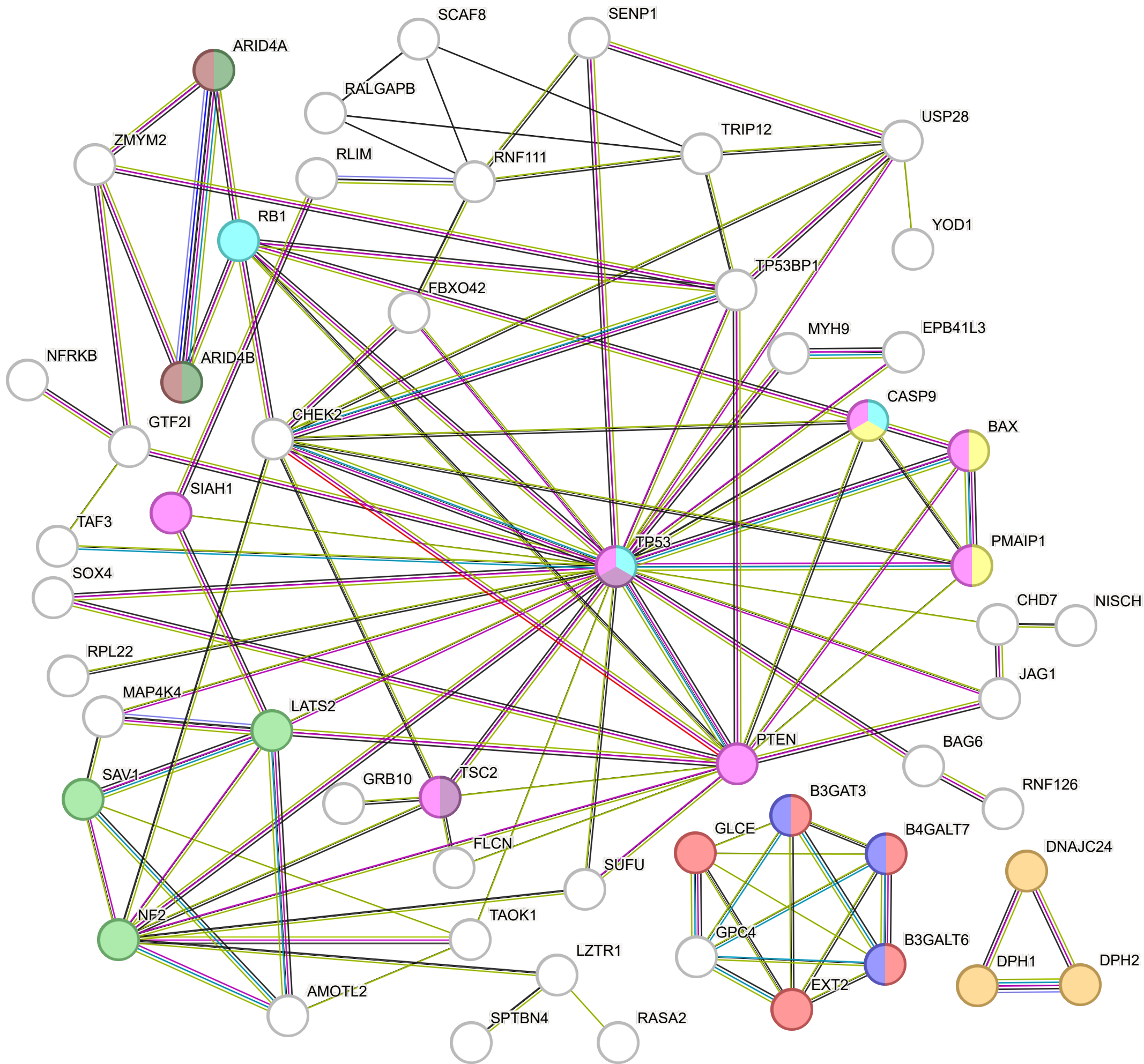

Supplement: Supplementary file 5 — Additional file 5. [file 10020_2025_1141_MOESM5_ESM.pdf]

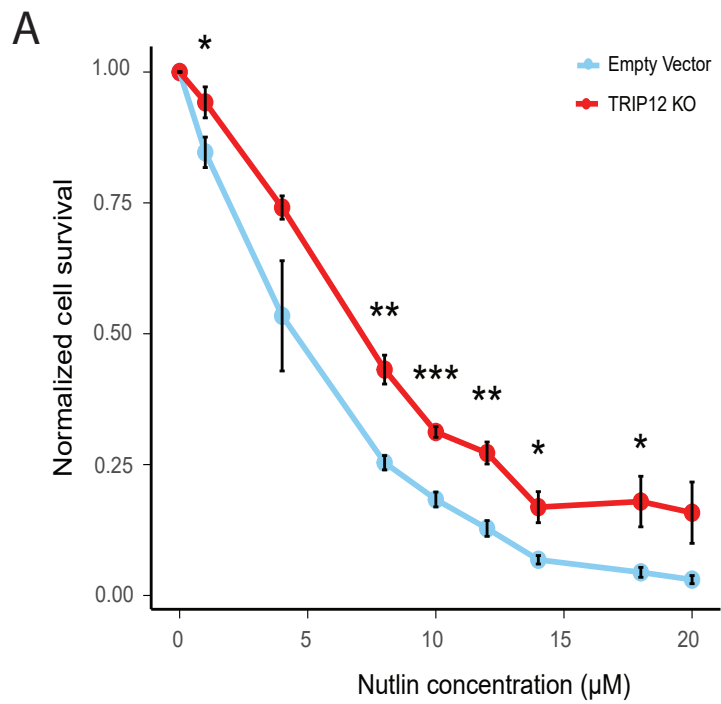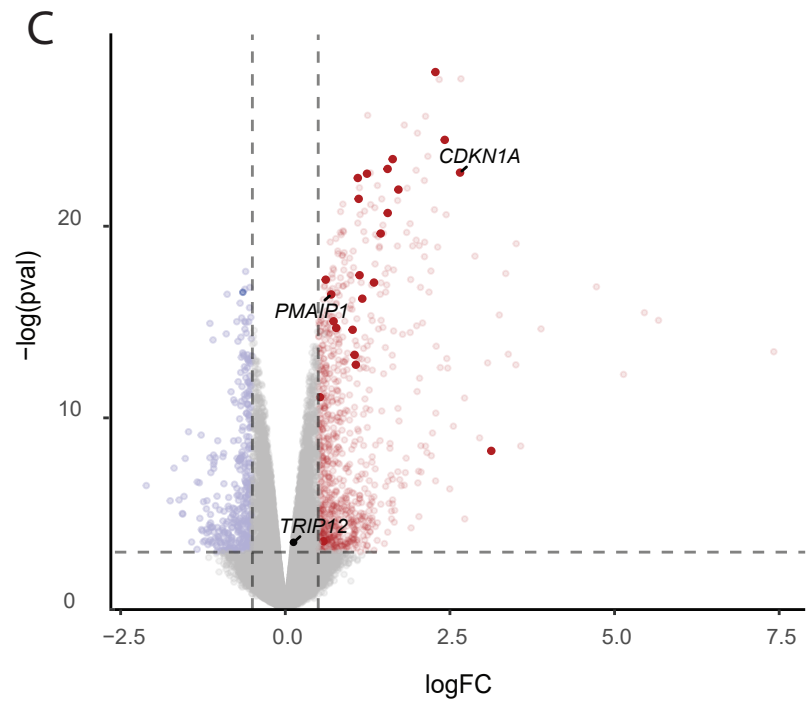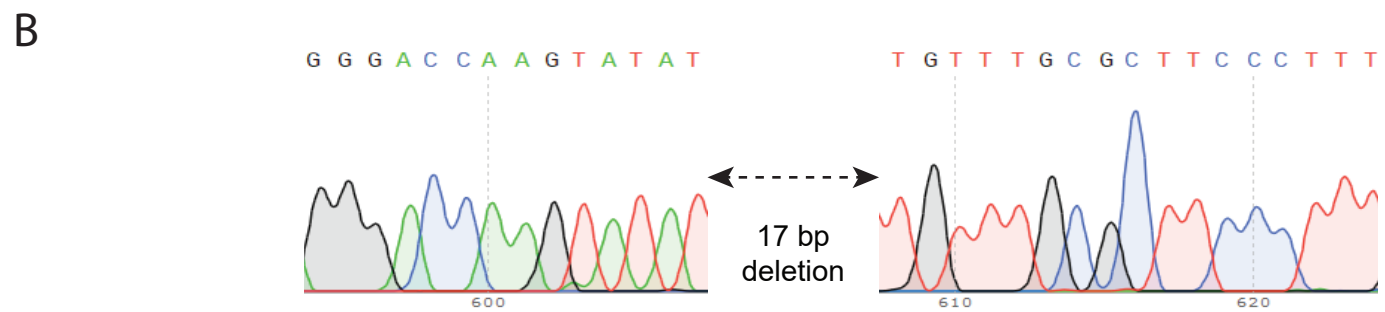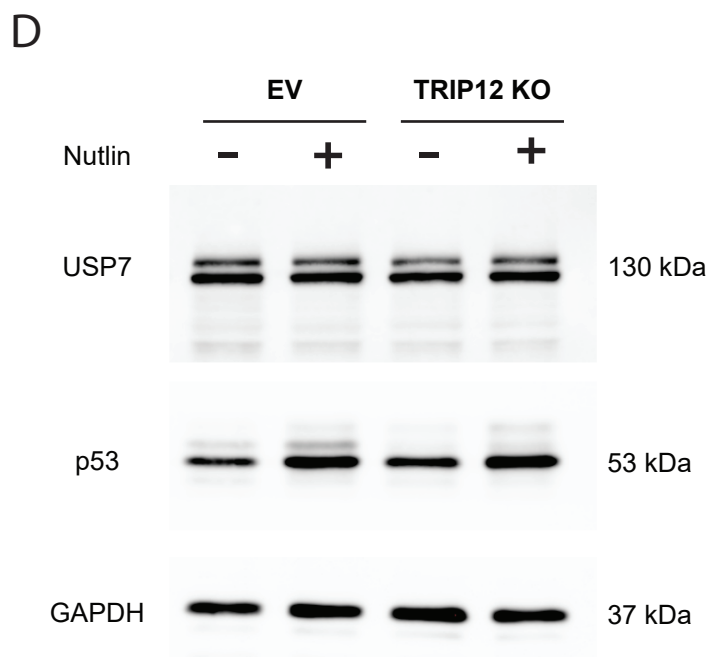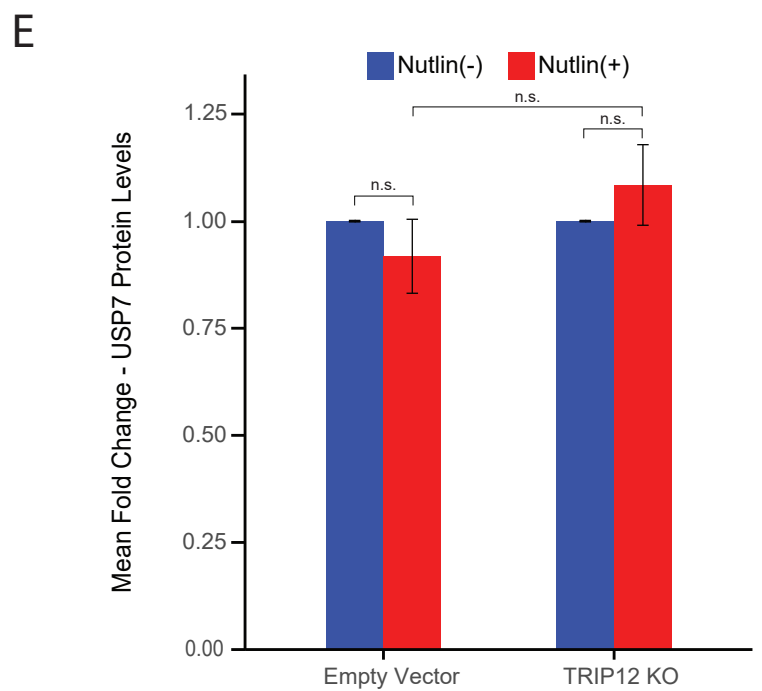

Supplement: Supplementary file 6 — Additional file 6. [file 10020_2025_1141_MOESM6_ESM.pdf]
