# Supplementary material for: Genome-wide screening in human embryonic stem cells identifies genes and pathways involved in the p53 pathway
Source: Mol Med. 2025 Mar 13;31:97. doi: 10.1186/s10020-025-01141-5 (PMC11907909; doi:10.1186/s10020-025-01141-5)
Supplement: Supplementary file 7 — Additional file 7. [file 10020_2025_1141_MOESM7_ESM.pdf]

Supplementary

Table S1

Table showing results of all genes that are statistically significant (FDR<0.05) in the screen.

Table S2

Table showing enriched KEGG and GO BP annotations in functional enrichment analysis by STRING of all genes that are statistically significant (FDR<0.05) and CS>1.5.

Table S3

Table displaying statistically significant results (p-value < 0.05) from the differential expression analysis of TRIP12 knockout clones and empty vector controls, following treatment with 6 μM Nutlin for 24 hours.

Figure S1

Calibration of Nutlin concentration for screen. Normalized survival as a function of time for different concentrations of Nutlin.

Figure S2

Protein-protein interaction network of all genes that are statistically significant (FDR<0.05) and CS>1.5 obtained from STRING.

Figure S3

A. TRIP12 KO clone sangers sequencing results showing the mutation. **B.** Normalized cell survival as a function of Nutlin concentration for TRIP12 KO mass culture cells compared to empty vector control cells. The graph represents the average of 3 biological repeats in experiment. Asterisks represent p-value of two-tailed paired t-test between each data point in knockout cells and WT cells. (*p-value < 0.05, **p-value < 0.01 and ***p-value<0.001). Error bars represent standard error of the mean. **C.** Volcano plot demonstrating -log(p-value) and logFC of differential expression analysis of non-treated vs Nutlin-treated (6 μM, 24 hours) samples of empty vector control cells. Horizontal dashed line indicates p-value=0.05. Vertical dashed lines mark logFC of -0.5 and 0.5. **D.** Western blot analysis of changes in USP7 and p53 protein levels in empty vector control and TRIP12 KO clone cells following treatment with 4 μM Nutlin for 6 hours. **E.** Mean fold change quantification of western blot results of USP7 levels relative to GAPDH. The graph represents the mean of 4 biological repeats, where in each repeat, the relative protein levels of the treated sample were divided by the corresponding untreated sample. Statistical significance was assessed using a two-tailed paired t-test comparing treated and untreated samples. “ns” indicates non-significant differences (p ≥ 0.05). Error bars represent the standard error of the mean.
